# Optimizing the HIV/AIDS informed consent process in India

**DOI:** 10.1186/1741-7015-2-28

**Published:** 2004-08-02

**Authors:** J Sastry, H Pisal, S Sutar, N Kapadia-Kundu, A Joshi, N Suryavanshi, KE Bharucha, A Shrotri, MA Phadke, RC Bollinger, AV Shankar

**Affiliations:** 1 Johns Hopkins University, Pune, India; 2 Institute for Health Management, Pachod (IHMP), India; 3 BJ Medical College and Sassoon Hospitals, Pune, India; 4 Directorate of Medical Education and Research, Mumbai, India; 5 Johns Hopkins University, School of Medicine, Baltimore, USA; 6 Johns Hopkins University, Bloomberg School of Public Health, Baltimore, USA

## Abstract

**Background:**

While the basic ethical issues regarding consent may be universal to all countries, the consent procedures required by international review boards which include detailed scientific and legal information, may not be optimal when administered within certain populations. The time and the technicalities of the process itself intimidate individuals in societies where literacy and awareness about medical and legal rights is low.

**Methods:**

In this study, we examined pregnant women's understanding of group education and counseling (GEC) about HIV/AIDS provided within an antenatal clinic in Maharashtra, India. We then enhanced the GEC process with the use of culturally appropriate visual aids and assessed the subsequent changes in women's understanding of informed consent issues.

**Results:**

We found the use of visual aids during group counseling sessions increased women's overall understanding of key issues regarding informed consent from 38% to 72%. Moreover, if these same visuals were reinforced during individual counseling, improvements in women's overall comprehension rose to 96%.

**Conclusions:**

This study demonstrates that complex constructs such as informed consent can be conveyed in populations with little education and within busy government hospital settings, and that the standard model may not be sufficient to ensure true informed consent.

## Background

It is estimated that nearly 7.2 million people in Asia and the Pacific region are now living with HIV/AIDS, one million of whom acquired the virus in 2002 [1]. Of these, more than 2.4 million are women (ages 15–45) [1]. India's national HIV prevalence rate of less than 1% offers little indication of the serious situation facing the country. An estimated 3.97 million people were living with HIV in India at the end of 2001, ranking it second only to South Africa in numbers of people infected. Although recent data suggest that prevention efforts directed at high-risk populations has resulted in greater HIV/AIDS knowledge and condom use [2,3], the prevalence of HIV/AIDS continues to rise. For HIV-positive pregnant women who have the additional risk of transmitting the disease to their unborn child, fewer prevention efforts are in place and are acutely needed. In some states in India such as Andhra Pradesh, Karnataka, Maharashtra, Manipur, Nagaland and Tamil Nadu prevalence rates are greater than 1% [1], underlining the need for well-planned and sustained interventions on a large scale.

In most industrialized countries, voluntary HIV counseling and testing services are a major component of HIV and AIDS control programs targeted toward pregnant women, although such programs are only now being advocated in developing countries [4,5]. Voluntary counseling and testing involves two basic components–an educational component on HIV/AIDS and an informed consent component related to an individual's choice to be HIV tested or not. The primary motivations for conducting voluntary HIV counseling and testing in lower risk populations such as pregnant women are that i) immediate knowledge of the woman's status can help reduce risk of HIV transmission to the unborn child; ii) if not infected, education at this point can help both the husband and wife to reduce risk behaviors to prevent transmission; and iii) testing of the partner can be encouraged [6]. Because voluntary HIV counseling and testing is the principal entry point for both prevention and care, it is critical that the content of the counseling is well understood and that procedures to ensure true informed consent are in place [7,8].

The basic ethical issues regarding consent may be universal to all countries. One of the potential limitations with the current approach is that consent has been reduced to a technical issue and is often removed from wider ethical considerations. The consent procedures required by international review boards include having to provide detailed scientific and legal information to participants. Although the intent is to increase knowledge, these messages may lose their meaning when administered within certain populations. The challenge then becomes one of how to balance the requirements of providing complete information while also obtaining true informed consent.

To begin establishing voluntary counseling and testing for pregnant women in India, the Indian National AIDS Control Organization (NACO) in 2001 started voluntary HIV counseling and testing for women attending government-run antenatal clinics in 11 sites throughout the country. This study was conducted in one of these sites, Pune, Maharashtra, in order to develop tools to enhance, standardize and improve the communication of messages during the group education and counseling (GEC) sessions, and to enhance the informed consent process.

## Methods

This study was conducted in the antenatal clinic of an urban government hospital in Pune. Eligible patients were newly registered pregnant women between 18 and 44 years of age who were less than 36 weeks gestation and had no physical or mental disabilities (as determined by a physician). After each eligible woman registered in the antenatal clinic and met with the attending physician, they were offered the opportunity to attend the HIV/AIDS GEC sessions.

The eight major topics covered in the education component of the GEC included general transmission modes of HIV, sexual transmission, mother-to-infant HIV transmission, precautions to avoid HIV transmission, ways through which HIV does not transmit, identification of HIV in an individual, detection of HIV in the body, and symptoms of AIDS. In addition, nine major topics related to HIV testing and informed consent were covered, including procedural risks of HIV screening, social risks of HIV screening, availability of the report, the right to say no, repercussions of refusing to take the test, confidentiality of the testing procedures, the right to consult others, the benefits of HIV screening, what one's signature means, specific procedures of the HIV ELISA test and, if applicable, specific procedures of the rapid HIV test.

### Baseline data

We conducted structured observations of 30 GEC sessions over the period from December 2000 to January 2001 to assess how well the information was being covered. The GEC sessions were chosen so that every clinic day (Monday-Saturday) and session time would be equally represented (there were, on average, four GEC sessions during the clinic hours of 9:30 am to 1:00 pm).

The structured observation tool included a comprehensive checklist of each of the specific points for each main topic covered during the GEC. A topic would be considered adequately covered if the counselor conveyed all the specific points for the topic. Overall session adequacy was assigned if at least 80% of all topics were presented adequately during the session.

To assess women's understanding of the key issues covered we interviewed 136 consecutively consenting women immediately after they attended one of the 30 sessions (89% of the total number of women who attended these sessions). Informed consent was obtained from each woman prior to the interview. After the interview, the women met briefly with an individual counselor, where they chose whether to have HIV testing and, if so, signed the appropriate informed consent form.

The women's understanding of the GEC sessions was determined through structured interviews containing 17 questions covering each of the main topics discussed in the GEC. Their answers were scored as either adequate or inadequate based on predetermined criteria.

Based in part on these observational and interview data we developed and enhanced the content of the GEC sessions. We also developed a comprehension test to evaluate women's understanding of key issues related to informed consent. The key enhancements to the GEC sessions were as follows:

• Areas with greater privacy for both group counseling and individual counseling were established.

• Posters illustrating the main topics were created and placed in the GEC rooms.

• Similar visuals were made into a flipchart and used by the counselor during individual counseling to reinforce the messages.

• All visuals developed included substantial input from the counselors.

• The visuals were simple, using bold colors and conveying only one message each.

• The posters were created to provide informational cues to the counselor to promote and maintain regularity and standardization in presentation.

• The counselors completed further training in the use of the visuals.

### Development of the comprehension test of key informed consent issues

Current standards for ethical treatment of human participants in a study do not require that participants demonstrate comprehension of the study prior to giving informed consent. However, given the volume and complexity of information conveyed during the GEC sessions, we felt it necessary to develop a concise yet complete comprehension test that could be used in HIV screening programs and adapted for future clinical trial enrollments.

The questionnaire was developed by choosing eight of the 17 questions from the previous questionnaire used during the baseline evaluation. The eight questions were identified by the field team of counselors, physicians, and behavioral scientists as key issues related to informed consent. The questionnaires were administered by the hospital counselor. Adequacy of women's understanding for any topic was determined if the woman correctly answered the question based on pre-determined criteria. If a woman scored adequately on at least 80%, or six out of eight, questions she became eligible to sign the informed consent form and proceed with the HIV screening. Prior to signing the form, the counselor had the opportunity to clarify any previously misunderstood issues with the woman.

The eight questions used to assess comprehension of informed consent issues were as follows:

1. What are the modes by which HIV germs are transmitted?

2. Can you say "No" to taking the HIV test?

3. What happens if you decide not to take the HIV test?

4. What do we mean by "the result of the test will be kept confidential"?

5. Do you have the right to consult your husband or other family members before taking the test?

6. What do you think are the benefits of finding out your HIV status?

7. What problems can a woman face on finding out her HIV-positive status?

8. What does your signature on the consent form mean?

### Post-intervention evaluation

After the enhancements to the GEC were put into place and the comprehension test developed, we conducted structured observations of 40 GEC sessions from May-June 2001. We performed the comprehension test on 224 women who attended one of these 40 sessions (89% of the total women attending). These same women were re-interviewed after the completion of individual counseling. The time between the interview after group counseling and the re-interview after individual counseling was, on average, one-two hours.

### Analysis

We compared the adequacy of GEC topic coverage prior to (baseline 30 GEC sessions) and after (40 GEC sessions) the inclusion of the enhancements. Also, improvements in the women's comprehension were assessed through a comparison of their responses to the eight key informed consent questions before GEC enhancements (*N = 136*) and after (*N = 224*). In addition, we examined the added improvements in knowledge when the second group of women (*N = 224*) were tested after their individual counseling. Analysis of the improvements in topic coverage and women's understanding of topics covered was determined by a chi-square test of proportions or Z-scores (for changes in knowledge in the same population) using SPSS version 10 [9].

This research was approved by the Institutional Review Boards (IRB) at Johns Hopkins University, Baltimore, USA, the local IRBs in Pune, the local medical institutions involved in the research and the National Institutes of Health, USA, which funded this study.

## Results

### Coverage of topics in GEC sessions

Topics related to informed consent and HIV testing were not well covered during the GEC sessions. Only three topics–availability of a report, the right to say no, and the benefits of screening–were covered in 90% or more of the sessions. The procedural and social risks of HIV screening, the right to consult others, and confidentiality were discussed in less than 10% of the sessions. The impact of refusing to be screened and what a signature means were discussed in 40% and 70% of the sessions, respectively.

### Coverage of informed consent issues with the use of visuals

Focusing only on the eight key issues related to understanding informed consent (see previously listed questions), we compared topic coverage before (Group A) and after (Group B) the improvements were made to the GEC sessions (Table 1). With the use of visuals, there were improvements in coverage in nearly all topic areas. The coverage of "the meaning of the signature" increased significantly from 70% to 90% (*P < 0.01*). Coverage of "social risk" during sessions showed a statistically significant increase from 7% to 23% (*P < 0.05*), yet the overall coverage was still very low. Similarly, the coverage of "the right to consult others" showed a statistically significant increase (*P < 0.001*), however, it was still only covered in 35% of the sessions. Non-significant improvements occurred in the area of "consequences of refusal", increasing from 40% to 55%. Overall, the use of visuals resulted in a statistically significant improvement in adequacy of coverage of the counselling sessions from 30% to 68% of all GEC sessions.

### Women's understanding of informed consent issues with the use of visuals

Table 2 shows both women's topic-specific and overall comprehension of informed consent issues. We compare improvements in comprehension in two groups of women, Group A (without use of visual) and Group B (with use of visuals). We then examine improvements in comprehension in the same group of women (Group B), after enhanced group counselling and with the addition of enhanced individual counselling. There was significantly better understanding in three critical areas of appropriate informed consent when visuals were used, namely, "the right to refuse", "consequences of refusal", and "the meaning of the signature." "The right to refuse" rose from 54% at baseline to 79% and 96% (*P < 0.01*) with enhanced group counseling and enhanced individual counseling, respectively. It should be noted that this topic was well covered in all sessions with or without the addition of visual aids. On the other hand, the discussion of "social risk if found to be HIV-positive" or "the right to consult others" was rarely covered in any of the counseling sessions. In spite of this, a considerable majority of women understood both these issues. Women's understanding of "consequences of refusal" showed marked improvements with use of visuals, rising from 19% at baseline to 75% with enhanced group counselling (*P < 0.01*) and further to 96% (*P < 0.01*) with enhanced individual counselling.

Overall, based on adequately answering six out of eight questions, women's knowledge of informed consent topics in enhanced group counselling improved dramatically, from 38% to 72% (*P < 0.01*). With the addition of individual counselling with visuals, women's overall understanding showed a statistically significant increase in all eight aspects of informed consent, and an overall increase from 72% to 96% (*P *< 0.01).

## Conclusions

There were two primary objectives of this study. The first was to enhance the communication of key concepts within the HIV/AIDS GEC setting and the second was to develop a simple tool to evaluate women's comprehension of informed consent issues.

Many international health education and prevention programs have developed and tested creative and culturally appropriate communication strategies to provide information on issues as sensitive and diverse as family planning and changing defecation behaviors. However, innovation in, and evaluation of, the process of delivering the often complex information necessary for informed consent has been limited [10]. The use of simplified visuals and text as a means of communicating messages to populations with little or no literacy has commonly been used to convey information on family planning, health, nutrition and public health activities [11,12]. However, such visuals must be developed with the specific population in mind and by community members in order to ensure their positive impact on understanding [13].

There is very little evidence demonstrating that individuals in resource-poor countries cannot understand the basics of research design or biomedical treatment options just because they have little education or different views about health and illness. It may be difficult to communicate the purposes, conditions and risks of research, but the difficulty of doing so should not detract from the importance of obtaining individual informed consent. In fact, several researchers [10,14] have established that, with some effort, acceptable levels of information can be communicated. This study indicates that the full informed consent process (including both group and individual counselling), when combined with enhanced education and counselling materials, can lead to excellent comprehension of informed consent issues.

The dramatic improvement we found in the women's comprehension of informed consent issues, despite their varying socioeconomic and educational backgrounds, is encouraging. Caution should be exercised, however, in interpreting the improvements in comprehension as being a result of the individual counseling and visuals. The second interview was done within one-two hours of the first, and the women may have had time to reconsider their responses to the previous questionnaire.

The major limitation in this study was the experimental design, in that we did not have the opportunity to measure the impact of individual counseling without the use of visuals. This was mainly because during baseline data collection we were interested in comprehension immediately after the GEC session; interaction with the counselor for individual counseling directly following the GEC was very brief and, therefore, was not expected to have improved women's knowledge considerably. Moreover, by the time we tested the visuals we had developed, the counselors had gained a lot more experience in HIV counseling and the women might have received more information from various media about HIV.

Despite these limitations, we can assert from our data that simple didactic group education on HIV/AIDS and testing issues is not sufficient to help women in this setting to understand the complexities of informed consent for HIV testing. The use of visuals in the form of posters and flipcharts provided structure and uniformity to the GEC sessions, thereby reinforcing the messages for these women and enhancing the overall informed consent process.

Obtaining proper informed consent in the case of HIV screening is not a discrete action, but a process that can be enhanced through effective communication, repetition and reflection. Although the intent is to increase knowledge, information regarding informed consent may lose its meaning when administered within certain populations. The time and the technicalities of the process itself may intimidate women in societies where literacy and awareness about medical and legal rights is low. It has been argued that complicated concepts conveyed in a consent form, often to fulfill the requirements of funding agency for institutional and policy purposes, may in themselves be unethical and, indeed, pose the biggest barrier to the informed consent process [15]. It should also be recognized that implementing counseling and informed consent procedures is considerably more difficult in certain settings in India where facilities, supplies, personnel, and time are at a premium. In addition, educational levels for women in this population are low, where 36% have a primary school education or less and another 33% are illiterate [16].

Generally, for HIV screening, an individual reads or is read a prepared statement that includes detailed scientific and legal information on the aims and biological significance of the test, the risks and benefits of testing, and the individual's rights. The participant is expected to understand the main components of what is written within the document and make an autonomous decision on whether or not to be tested for HIV. This is further complicated by the fact that, in some studies, the original consent document is composed in English and then translated nearly verbatim into the local language, making the communication of already complex topics even more difficult.

Typically, informed consent for pregnant women in most Indian hospitals and clinics is for operative procedures such as cesarean section or laparotomy. It is usual for the doctor or resident on duty to put down in his or her own handwriting the text of the consent on a patient's case papers. The content of this consent gives blanket permission to the hospital and doctors to undertake all procedures on the patient that are indicated in order to maintain the good health of the mother and her fetus, while at the same time absolving the attending physician and hospital of any blame in the event of a mishap. This is signed (or a thumbprint given) by the patient, her husband or an accompanying relative, and is generally considered to serve as legal consent; therefore, it is not interpreted as voluntary. Most often, due to time constraints, very little is explained to the patient about the procedure, risks, and benefits, or what her signature actually means. As found in other regions in India, there is a general perception by clinicians and other healthcare workers that women are "unable" to understand any of the procedures even if explained, because they are illiterate or have no medical background [17,18]. It is implicit in the physician-patient relationship that any treatment or procedures recommended by the physician will benefit the patient [19-21]. By de-mystifying the content and process of informed consent through standardization with structured visual cues and reiteration, we feel that these difficulties could be overcome.

In creating the modifications to the GEC, we focused on improving communication of those concepts that were the most unfamiliar to these women. For example, for most women in India, there is relatively little sense of autonomy [22]. For many, the woman's role is defined first by her father; after marriage, her husband's decision-making and the wishes of his parents or the elders in his home prevails. For such a woman, when an incurable disease like HIV/AIDS is presented to her in terms of her "autonomy" and "power" with reference to the disease, this could be confusing and even frightening. The prevailing practice of obtaining familial input on such decisions was demonstrated through women's knowledge of this topic despite the fact that it was hardly mentioned during the counseling sessions. On the other hand, our data show that by enhancing the GEC and reinforcing its messages through individual counseling, a significantly greater number of women can correctly understand the idea of their "right to refuse", indicating that even complex constructs such as autonomy can be conveyed. The understanding of the "meaning of the signature" was clearly enhanced with the use of visuals, because the improvements in women's knowledge directly followed the increase in coverage during the counseling sessions.

Clearly, some of the concepts related to informed consent may already be understood by these women. Women's understanding of "consequences of refusal" showed marked improvements in the second group of women studied. Although some of this improvement may be attributed to the individual counselling and visuals, the fact that only 55% of the GEC sessions actually adequately covered this topic indicates that other factors may have contributed to the women's knowledge of this topic.

This process of refining and evaluating the informed consent process can benefit the clinic and research settings in both developed and developing countries. Data from clinics in the USA, Belgium, and France report that even under well suited environments, informed consent for HIV screening was generally only obtained in about 70–85% of cases, and documentation of consent was substantially less [23,24]. In research settings, studies suggest that, despite having signed a consent form, participants may not fully understand critical aspects of research participation or their individual rights [25,26].

We have modified the standard model of informed consent by adapting it to suit the population that was served, and have documented subsequent improvements in patient understanding of the informed consent process. This study shows that culturally appropriate enhancement of the standard informed consent process moves the process towards its goal of being one that is truly informed and voluntary. Thus it not only fulfills the ethical requirements, but, most importantly, helps to assure that women's rights are preserved. This last point is critical because previous research has pointed out that, although women may be fully informed, they still may not feel their choice is fully voluntary [14]. We suggest that the current requirements of informed consent procedures are inadequate and that it should be a process that communicates information in an effective manner, allows for reiteration of information and includes an evaluation of the woman's knowledge prior to signing the informed consent document.

In an effort to allow all interested organizations involved in HIV counseling and testing to use or modify our visuals for their own programs, we are have created a downloadable version available for public access at our website . It is hoped that these types of visuals will become an integral part of all voluntary counseling and testing programs throughout India and elsewhere.

## Competing interests

None declared.

## Author's contributions

JS was involved in developing the research and visuals, implementing the study and preparing the manuscript. HP participated in the design of the visuals and in the analysis and coordination of the study. SS and AJ were involved in data collection and analysis. NK-K participated in the design of the visuals and in the analysis of the study. NS participated in the design of the study and the visuals. KEB, AS and MAP participated in the design and assisted in the implementation of the study. RCB assisted in all aspects of the study design and implementation. AVS participated in the design, conducted the analysis and wrote the manuscript. All authors read and approved the final manuscript.

## Pre-publication history

The pre-publication history for this paper can be accessed here:



## Figures and Tables

**Table 1:** Coverage of selected components of informed consent during counseling sessions, with and without the use of visuals

**Topic covered**	Coverage in 30 group education sessions **without **visuals (Group A)		Coverage in 40 group education sessions **with **visuals (Group B)		Chi square ***χ***^2 ^statistic and significance level
	*N*	%	*N*	%	
**Mother-to-infant HIV transmission**	30	100	40	100	___
**Confidentiality**	30	100	38	95	0.2
**Right to refuse**	29	97	39	98	0.0
**Consequences of refusal to test**	12	40	22	55	1.4
**Social risk**	2	7	9	23	4.0*
**Benefits of HIV testing**	30	100	39	98	0.0
**Meaning of signature**	21	70	36	90	6.8**
**Right to consult**	0	0	14	35	12.6***
**80% coverage in sessions**	9	30	27	68	13.4***

* = P < 0.05, ** = P < 0.01, *** = P < 0.001

**Table 2:** Relative improvement in women's understanding of components of informed consent after use of visuals in group education and counseling (GEC) and individual counseling (IDC)

**Topic covered**	Group A: Women's understanding after GEC **without **visuals (posters) (*N = 136*)		Group B: Women's understanding after GEC **with **visuals (posters) (*N = 224*)		Chi square ***χ***^2 ^statistic Group A *vs *Group B	Group B: retested after IDC **with **visuals (flipcharts) (*N = 224*)		Z Score statistic Group B (GEC) *vs *Group B (GEC+IDC)
	*N*	%	*N*	%		*N*	%	
**Mother-to-infant HIV transmission**	128	94	213	95	0.03	221	99	2.17**
**Confidentiality**	130	76	174	78	0.09	219	98	6.47***
**Right to refuse**	73	54	176	79	23.43***	215	96	5.52***
**Consequences of refusal to test**	26	19	167	75	102.35***	214	96	6.22***
**Social risk**	86	63	153	68	0.76	194	87	4.63***
**Benefits of HIV testing**	39	29	88	39	3.72	155	69	6.35***
**Meaning of signature**	80	59	169	75	10.19***	215	96	6.21***
**Right to consult**	123	90	203	91	0.02	216	96	2.49**
**Overall adequate understanding of women (6/8 questions)**	52	38	162	72	39.38***	216	96	7.02***

* = P < 0.1, ** = P < 0.05, *** = P < 0.01
